# The Potential Key Role of the NRF2/NQO1 Pathway in the Health Effects of Arsenic Pollution on SCC

**DOI:** 10.3390/ijerph19138118

**Published:** 2022-07-01

**Authors:** Qianlei Yang, Rui Yan, Yuemei Mo, Haixuan Xia, Hanyi Deng, Xiaojuan Wang, Chunchun Li, Koichi Kato, Hengdong Zhang, Tingxu Jin, Jie Zhang, Yan An

**Affiliations:** 1 Department of Toxicology, School of Public Health, Jiangsu Key Laboratory of Preventive and Translational Medicine for Geriatric Diseases, Medical College of Soochow University, Suzhou 215123, China; qlyang@suda.edu.cn (Q.Y.); 15895572995@163.com (R.Y.); 20184047006@stu.suda.edu.cn (H.X.); 20214047009@stu.suda.edu.cn (X.W.); zhangjie_78@suda.edu.cn (J.Z.); 2 Physical Examination Department, Center for Disease Control and Prevention of Suzhou Industrial Park, Suzhou 215100, China; 0219501041@163.com; 3 Shanghai Municipal Center for Disease Control and Prevention, Shanghai 200336, China; dhysuda@126.com; 4 Changzhou Wujin District Center for Disease Control and Prevention, Changzhou 213164, China; 20144247028@stu.suda.edu.cn; 5 Laboratory of Environmental Toxicology and Carcinogenesis, School of Pharmacy, Nihon University, Chiba 274-8555, Japan; kato.koichi@nihon-u.ac.jp; 6 Department of Occupational Disease Prevention, Jiangsu Provincial Center for Disease Control and Prevention, Nanjing 210028, China; hdzhangjscdc@yeah.net; 7 Jiangsu Preventive Medicine Association, Nanjing 210009, China; 8 School of Public Health, The Key Laboratory of Environmental Pollution Monitoring and Disease Control, Ministry of Education, Guizhou Medical University, Guiyang 550025, China

**Keywords:** arsenite pollution, health effect, squamous cell carcinoma, NRF2/NQO1 pathway, cell proliferation, malignant transformation

## Abstract

Arsenic is widely present in nature and is a common environmental poison that seriously damages human health. Chronic exposure to arsenic is a major environmental poisoning factor that promotes cell proliferation and leads to malignant transformation. However, its molecular mechanism remains unclear. In this study, we found that arsenite can promote the transformation of immortalized human keratinocyte cells (HaCaT) from the G0/G1 phase to S phase and demonstrated malignant phenotypes. This phenomenon is accompanied by obviously elevated levels of NRF2, NQO1, Cyclin E, and Cyclin-dependent kinase 2 (CDK2). Silencing the NRF2 expression with small interfering RNA (siRNA) in arsenite-transformed (T-HaCaT) cells was shown to reverse the malignant phenotype. Furthermore, the siRNA silencing of *NQO1* significantly decreased the levels of the cyclin E-CDK2 complex, inhibiting the G0/G1 to S phase cell cycle progression and transformation to the T-HaCaT phenotypes. Thus, we hypothesized that the NRF2/NQO1 pathway played a key role in the arsenite-induced malignancy of HaCaT cells. By increasing the expression of Cyclin E-CDK2, the NRF2/NQO1 pathway can affect cell cycle progression and cell proliferation. A new common health effect mechanism of arsenic carcinogenesis has been identified; thus, it would contribute to the development of novel treatments to prevent and treat skin cancer caused by arsenic.

## 1. Introduction

As the twentieth element of the Earth’s crust, arsenic is most commonly found in the form of sulfides in nature. Arsenic is released into the environment from natural sources as a result of natural phenomena, such as dust storms, volcanic eruptions, geothermal/hydrothermal activity, and forest fires [1]. Arsenic can interact with oxygen and other molecules of nature to form different compounds. Therefore, it is extremely commonplace in the daily life of humans [2]. Arsenic is widely used in the manufacture of industrial and agricultural products such as semiconductors, glassware, alloys, wood preservatives, and pesticides and in medical treatments for diseases such as syphilis, rickets, amoebic dysentery, and leukemia [3,4]. These human activities also have the potential to cause severe arsenic pollution in the environment. Arsenic contamination of drinking water and soil has become an international and regional problem over the past two decades. A survey of arsenic concentrations in groundwater in eastern Wisconsin, United States, found naturally occurring arsenic concentrations in groundwater of more than 12,000 μg/L, which is much higher than the U.S. Environmental Protection Agency (EPA) and WHO standard/baseline concentrations for arsenic in drinking water [5]. In other countries and regions, such as Argentina, Bangladesh, Chile, China, Mexico, India, Thailand, and Taiwan, the health effects of arsenic contamination in groundwater have become one of the major public health problems [3,6].

Arsenic is a common environmental toxin with toxic and teratogenic effects in many metazoans [7,8,9]. As water is the primary polluting medium for arsenic, aquatic organisms bear the brunt of arsenic [10,11,12]. In addition, humans have the highest risk of arsenic contamination due to the food chain. It is estimated that nearly hundreds of millions of people are exposed to the threat of arsenic compounds in drinking water and food worldwide [13,14,15]. The main source of human arsenic intake comes from water and food, with an average daily intake of approximately 50 μg [3]. The human arsenic intake from air, water, and soil is usually much smaller. However, these environmental media also deserve particular attention in areas contaminated with arsenic. In certain workplaces where arsenic compounds are produced and used, workers may be exposed to greater concentrations of arsenic than in the natural environment [16,17,18]. Thus, arsenic exposure is considered the leading cause of global health problems today. In the Indian subcontinent, for instance, exposure to arsenic and having related diseases have reached alarming levels, where nearly 359 million people may be at risk [19]. Long-term exposure to arsenic can lead to a number of adverse health effects, including hyperkeratosis, jaundice, and vascular diseases [20]. However, the carcinogenic role of arsenic has brought more significant concerns in recent years, as arsenic-related cancers can be diagnosed in the skin, lungs, liver, and other malignant tumors [21,22,23,24]. Arsenic is now classified as a Group A carcinogen by the EPA and a Group I carcinogen by the International Agency for Research on Cancer (IARC). Among the cancers caused by arsenic, skin cancer was the first one that has been confirmed. As early as the late 1880s, White et al. reported one case of skin cancer caused by psoriasis treated with potassium arsenite [25]. Currently, skin cancer is commonly referred to as skin melanoma (MC) and non-melanoma skin cancer (NMSC), the latter being composed of basal cell carcinoma (BCC) and squamous cell carcinoma (SCC) [26]. Numerous epidemiological studies have shown that apart from ultraviolet (UV) radiation, arsenic is the most significant chemical damage factor related to SCC and BCC [27,28,29,30]. Unfortunately, the mechanics of arsenic-induced skin cancer are unclear, making treatment and control extremely difficult.

In most cases, the final outcome of cellular differentiation is antiproliferative and constitutes a barrier to the continued proliferation required for tumorigenesis. Therefore, the hallmarks and characteristics of cancer currently include the acquired capabilities for sustaining proliferative signaling, evading growth suppressors, resisting cell death, and enabling replicative immortality [31]. As the mechanisms of cell proliferation, malignant transformation, and carcinogenesis are complex, the Nrf2/NQO1 pathway is considered one of them. Nuclear erythroid factor 2 p45 (NRF2) is of primary importance in the alteration of cell homeostasis to respond in an adaptive manner to xenobiotic and oxidative stress [32]. The accumulation of studies has shown that NRF2 plays a significant role in cell proliferation, except for the regulation of intracellular redox homeostasis [33,34]. NRF2 has long been considered a cancer inhibitor, providing the rationale for cancer prevention strategies using NRF2 activators [35]. However, increased NRF2 activity has recently been observed in a wide range of cancers [36]. This deregulated Nrf2 condition provides several growth advantages to cancer cells [35]. Therefore, NRF2 has attracted great interest for its use in cancer therapy and diagnosis. NAD(P)H: quinine oxidoreductase 1 (*NQO1*) is one of the target genes regulated by the transcription factor NRF2 [37,38]. The function of NQO1 is widely regarded as a “cytoprotective agent” that protects cells from oxidative damage by inducing responses to various noxious stimuli [39,40]. As with NRF2, the accumulation of studies confirmed the high expression of the *NQO1* gene in solid tumor tissue in recent years [41,42,43]. The upregulation of NQO1 may contribute to the growth of cancer cells, especially in oxidative stress environments [44,45,46]. Furthermore, the negative expression of NRF2 and NQO1 provides a better prognosis for patients with non-small cell lung cancer (NSCLC) [47]. In fact, we have reported that the long-term exposure to arsenite induced NRF2 accumulation and enhanced proliferation in human bronchial epithelial cells (HBE) [48] and immortalized human keratinocyte cells (HaCaT) [44], and we have found that the upregulation of NRF2/NQO1 caused the proliferation and transformation of arsenite-induced HBE cells by increasing cyclin E-CDK2 and then affecting the cell cycle [48]. It is suggested that a novel mechanistic pathway for arsenite carcinogenesis, the NRF2/NQO1 pathway, is involved in the dislocation of cell cycle progression in arsenite-induced malignancies. Nevertheless, this mechanism has not been confirmed in the study of arsenic-induced skin cancer.

In this study, we aimed to elucidate the mechanism of the NRF2/NQO1 pathway in mediating arsenite-induced malignant phenomena in HaCaT cells. To achieve our study objective, we analyzed the mechanism of dynamic changes in cell proliferation, NRF2, NQO1, cell cycle-related proteins, and cyclin-dependent kinases with arsenic-induced neoplastic transformation in HaCaT cells. In addition, we examined the actions of NRF2/NQO1 in arsenite-transformed HaCaT cells (T-HaCaT). We hope this study will lead to a greater understanding of arsenite skin toxicity, which may provide more possibilities for clinical applications for SCC.

## 2. Materials and Methods

### 2.1. Cell Culture and Exposure to Arsenic

HaCaT cells were developed by Boukamp et al. as a line of spontaneously immortalized human epithelial cells. They are immortal, nontumorigenic, and retain most of the characteristics of normal human keratinocytes (NHKs). HaCaT cells offer a suitable and stable model for keratinization studies. Moreover, this line could be reproducibly transfected with the activated human Ha-ras oncogene. Selected clones gave rise to highly differentiated benign epidermal cysts and/or squamous cell carcinomas in nude mice [49]. Thus, the HaCaT cell line is ideal for SCC studies. In this study, HaCaT cells were generously supplied by Prof M.Y (Dalian Medical University, Dalian, China). The cells were incubated at 37 °C in a humidified 5% atmosphere in Dulbecco’s modified Eagle’s medium (DMEM; Life Technologies/Gibco, Grand Island, NY, USA) with 10% fetal bovine serum (FBS; Hyclone, Thermo Scientific, Waltham, MA, USA), 100 U penicillin/mL, and 100 µg of streptomycin/mL. To create cellular patterns for the chronic arsenic exposure model with final concentrations of DMEM with 0.0 (control) or 1.0 µM sodium arsenite (NaAsO_2_; Merck Drugs & Biotechnology, Darmstadt, Germany, purity 99.0%), HaCaT cells were cultured for 35 passages (approximately 18 weeks). The experimental control cells were HaCaT cells exposed to 0.0 μM NaAsO_2_ for passage 0, while the transient matched control cells were exposed to 0.0 μM NaAsO_2_ at other times. T-HaCaT cells were defined as 35-passage HaCaT cells exposed to arsenite based on our previous study [44]. Analytical and quality-assured reagents were used in all experiments.

### 2.2. Proliferation of Cells Assay

To determine whether exposure to 0.0 or 1.0 μM NaAsO_2_ at various passages (0, 1, 7, 14, 21, 28, and 35 passages) of HaCaT cells would induce transformation, cellular proliferation changes were measured. As instructed by the manufacturer, the cell proliferation assay was measured by a 3-(4,5-dimethyl-2-thiazolyl)-2,5-diphenyl-2-H tetrazolium bromide (MTT) kit. In brief, before 10 μL of MTT labeling reagent was added, 50,000 cells/mL were washed with phosphate-buffered saline (PBS), seeded onto 96-well plates, and incubated at 37 °C with 5% CO_2_ for 24 h. Afterward, 100 μL of solubilization solution was added 4 h later. Finally, a microplate scanner was used to measure blue formazan from viable cells at 570 nm in accordance with the manufacturer’s protocol. The experiment was repeated three times.

### 2.3. Flow Cytometry Analysis of the Cell Cycle

The cell cycle was detected by the cell cycle test kit. As per the manufacturer’s instructions, synchronizing the cells before exposure required 48 h of culture in DMEM with 0.5% FBS. After continuous exposure to arsenite over a period of time, HaCaT cells were harvested at different passages, collected with 0.25% trypsin without EDTA, and washed twice with 4 °C pre-cooled PBS. The cells were left at −20 °C overnight with the addition of 1 mL of 70% pre-cooled ethanol. After two rounds of washing in cold PBS, the cells were stained for 30 min with 20 μg/mL of PI (Beyotime Institute of Biotechnology, Haimen, China) and 200 μg/mL of ribonuclease A (RNaseA, Beyotime Institute of Biotechnology, Haimen, China) diluted in PBS. Following the above processing, the data were analyzed using cell cycle analysis software based on flow cytometry (Beckman Coulter FC500, Brea, CA, USA) for detection. The experiment was repeated three times.

### 2.4. Western Blots

The experimental process was as follows: After three washes with ice-cold PBS and centrifugation at 16,000× *g* for 10 min at 4 °C, whole cell extracts were obtained using a cell lysis buffer for Western blot and Immunol precipitation (IP) (Beyotime Institute of Biotechnology, Haimen, China) with 1.0% phenylmethanesulfonyl fluoride (PMSF; Beyotime Institute of Biotechnology, Haimen, China), and the protein fractions were quantified using a BCA Protein Assay Kit (Beyotime). Protein lysates were subjected to sodium polyacrylamide dodecyl sulphate gel electrophoresis (SDS-PAGE) and transferred to polyvinylidene difluoride membranes (Millipore, Bedford, MA, USA) in equal amounts. The membranes were blocked with 5% skim milk (Yili Industrial Group Limited by Share Ltd., Hohhot, China) for 1 h and then incubated with the appropriate primary antibodies overnight at 4 °C. The following primary antibodies were used in this research: NRF2 (sc-13032) (dilution 1:500), NQO1 (sc-32793) (dilution 1:500), and CDK2 (sc-6248) (dilution 1:500) were purchased from Santa Cruz Biotech (Santa Cruz, CA, USA); Cyclin E (WL01072) (WB 1:750) and Cyclin D1 (WL01435a) (WB 1:750) were acquired from Wanlei Organic Company (Shenyang, China); Cycline A (RLT1167) (dilution 1:500) was acquired from Suzhou Ruiying Organic Company. The loading control was GAPDH (Beyotime Institute of Biotechnology, Haimen, China) (dilution 1:1000) or β-actin (sc-47778) (dilution 1:500) (Santa Cruz, CA, USA). Following three washes in TBST (Tween 20 Tris-buffered saline) saline solution, membranes were incubated with secondary antibodies conjugated with equine radish peroxidase (Beyotime). An improved chemiluminescence kit (Millipore, Bedford, MA, USA) and G: BOX Chemi XRQ (Syngene, Cambridge, UK) were used for the detection of immunoreactive proteins. To correct for changes in protein load between the different test groups, densitometry was used to quantify the plots and standardize them with GAPDH or β-actin. The results obtained relate to three autonomous experiments.

### 2.5. SiRNA Interference Assays

According to the manufacturer’s protocol, *NRF2* siRNA transfection and *NQO1* siRNA transfection were performed on the 35 passages (T-HaCaT) cells exposed to 1.0 μM arsenite to silence the *NRF2* and *NQO1* genes to further understand the roles of NRF2/NQO1 in our research. First, 2 × 10^5^ cells were seeded in 2 mL of antibiotic-free DMEM containing FBS and then added to each well of a 6-well plate 24 h before transfection. Next, the biphasic siRNA solution (solution A) and diluted transfection reagent (solution B) (sc-29528, Santa Cruz Biotechnology) were mixed with siRNA transfection medium (sc-36868, Santa Cruz Biotechnology) prior to siRNA in preparing a mix of transfection reagents. The above mixtures were incubated at room temperature for 15–45 min and then added to the cells and stirred evenly. The cells were incubated in a 37 °C 5% CO_2_ incubator for 5–7 h, and then 1 mL of DMEM containing 20% fetal bovine serum and 2% antibiotics was added without removing the transfection mixture. After incubation for 18–24 h, the cells were collected for analysis. Control siRNA, *NRF2* siRNA, and *NQO1* siRNA were purchased from Santa Cruz Biotechnology (Santa Cruz, CA, USA). HaCaT cells (passage 35), not exposed to arsenite, served as a negative control.

### 2.6. Wound-Healing (Cell Migration) Assay

The wound-healing assay is a method for studying the role of various experimental conditions in cellular migration and proliferation. It is simple and cost-effective and is one of the earliest methods to be used for targeted in vitro cell migration studies. To determine cell migration, wound healing experiments were conducted. The objective of the wound-healing test was to analyze the impact of NRF2 and NQO1 inhibition on cell migration. In short, HaCaT and T-HaCaT cells with or without *NRF2* siRNA and *NQO1* siRNA transfection were seeded in 6 cm dishes to form confluent monolayers. The cell monolayer was wounded using a 200 μL pipette tip and washed twice with PBS. Then, the wound width (0 h and the subsequent 24 h incubation period were photographed immediately after scratching. The tests were conducted in three petri dishes in triplicate, and the results are expressed as the relative scratch width based on the distance migrated relative to the original scratched distance. Cell migration rate = (the width of the wound at 0 h-the width of the wound at 48 h)/the width of the wound at 48 h.

### 2.7. Anchor-Independent Growth Experiment

Normal cell proliferation requires the costimulation of growth factor signaling and cell adhesion, but an important feature of phenotypically transformed cells is anchor-independent growth. To determine the growth of cells independent of the anchor, a soft agar cloning assay was used. Sweet AGAR plates were prepared with 0.70% agarose underlay in DMEM supplemented with 10% FBS in a 35 mm (diameter) container. To observe the growth capacity of colonies on soft AGAR, cells were cultivated in three copies at a density of 5 × 10^3^ in 2 mL of 0.35% agarose overlaid with 0.70% agarose in the same medium. Cells were incubated at 37 °C in a humidified atmosphere at 5% with a routing replenished of medium every three days. After 3 weeks, colonies of more than 30 cells were examined under a microscope and photographed under a dissective microscope.

### 2.8. Statistical Analysis

Data analysis was performed using SPSS software (version 20.0, SPSS Inc., Chicago, IL, USA). All experimental data were tested for homoscedasticity, and differences between groups were assessed using one-way analysis of variance (ANOVA). Pairwise comparisons of sample means with uniform variance were performed by ANOVA using the SNK test and Tamhane’s T2 test for nonuniform variance. The test results were statistically significant at *p* < 0.05. Data from the triplicate experiments reported as the mean ± standard deviation (SD) were analyzed.

## 3. Results

### 3.1. Malignant Phenotypes Were Induced in HaCaT Cells with Continuous Arsenic Exposure

**Figure 1:**
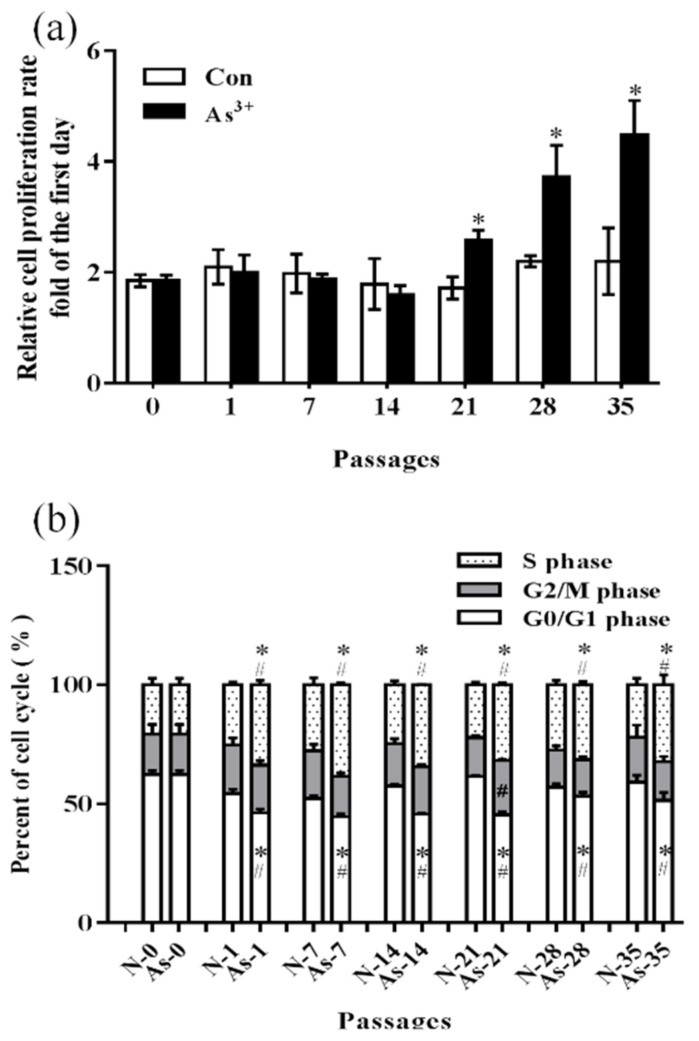
Changes in cell proliferation and cell cycle in HaCaT cells exposed to 1.0 μM arsenite or without arsenite exposure for different passages. (**a**) Change in the cell proliferation rate in HaCaT cells. (**b**) Change in the cell cycle in HaCaT cells. Error bars are the mean ± SD (*n* = 3). * Compared with the passage-matched control group, *p* < 0.05; ^#^ Compared with the experimental control group, *p* < 0.05.

To drive cells toward a malignant phenotype, normal HaCaT cells were exposed to 0.0 or 1.0 μM NaAsO_2_ for 18 weeks (35 passes). As a result, the proliferation rate in the treatment group (1.0 μM NaAsO_2_) was significantly higher at passages 21, 28, and 35 than that in the passage-matched control group (0.0 μM NaAsO_2_) and showed a trend of increasing passage by passage (*p* < 0.05) (Figure 1a). Furthermore, the ratio of G1 phase cells was significantly decreased, and the ratio of the S phase was significantly increased in the treatment group from passage 1 to 35 (*p* < 0.05) (Figure 1b), which indicated that arsenite can promote the transformation of HaCaT cells from the G0/G1 phase to S phase. The data demonstrated malignant phenotypes (T-HaCaT) in cells processed by NaAsO_2_.

### 3.2. Decreased Cell Cycle-Related Proteins and NRF2 and NQO1 in HaCaT Cells Exposed to Arsenite

**Figure 2:**
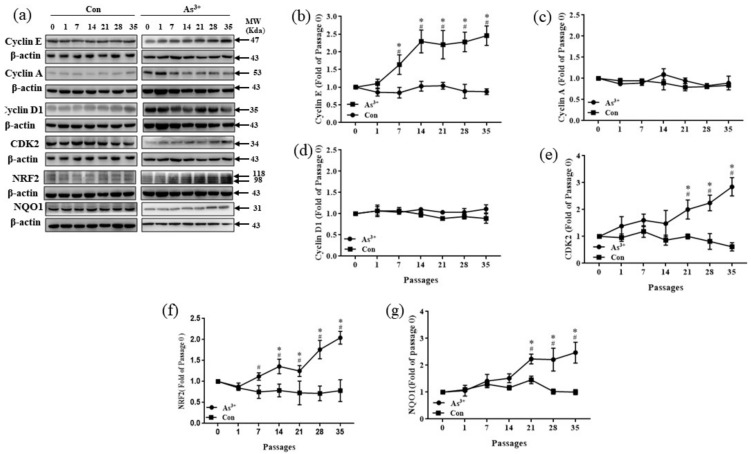
Relative protein expression and quantification of changes in NRF2, NQO1, and cell cycle-related proteins in HaCaT cells exposed to 1.0 μM arsenite or without arsenite exposure for different passages. (**a**) Protein bands. (**b**) Relative protein levels of Cyclin E. (**c**) Relative protein levels of Cyclin A. (**d**) Relative protein levels of Cyclin D1. (**e**) Relative protein levels of CDK2. (**f**) Relative protein levels of NRF2. (**g**) Relative protein levels of NQO1. Error bars are the mean ± SD (*n* = 3). * Compared with the passage-matched control group, *p* < 0.05; ^#^ Compared with the experimental control group, *p* < 0.05.

Cyclin proteins, including A, B, D, E, G, and H, collaborate with their cyclin-dependent kinases. They act as the nucleus of the cell cycle process [50]. Cyclin and cyclin kinases were measured in HaCaT cells as indicators to assess changes in the cell cycle in arsenite-induced malignant transformation in this study. Compared with the passage-matched control groups, Cyclin E was significantly increased after passage 7 and showed an upward trend after continuous exposure to 1.0 μM NaAsO_2_ (*p* < 0.05) (Figure 2b–d). Moreover, the protein expression of cyclin-dependent kinase CDK2 showed a significant increase after 21 passages in HaCaT cells and increased with each passage; however, similar changes were not observed in the control groups (*p* < 0.05) (Figure 2e). No significant differences were observed between the other cyclin and the cyclin-dependent kinases with the control groups. These results suggest that arsenite-exposed cell proliferation promotes the progression of the cell cycle from the G0/G1 phase to S phase through an increase in cyclin E-CDK2, thereby causing the malignant transformation of HaCaT. Changes in NRF2 and NQO1 were also measured. During the 1.0 μM NaAsO_2_-induced malignant transformation in HaCaT cells, the elevated expression levels of NRF2 and NQO1 increased. As shown in Figure 2f,g, compared to the control groups, the protein level of NRF2 expression increased significantly after seven passages and displayed an upward trend. As a target of NRF2, the protein expression of NQO1 was upregulated after passage 21 (*p* < 0.05). These results indicate that the protein level of NRF2 and its target gene *NQO1* were increased by arsenite exposure.

### 3.3. NRF2-Mediated Cellular Proliferation in Arsenite-Exposed HaCaT Cells

**Figure 3:**
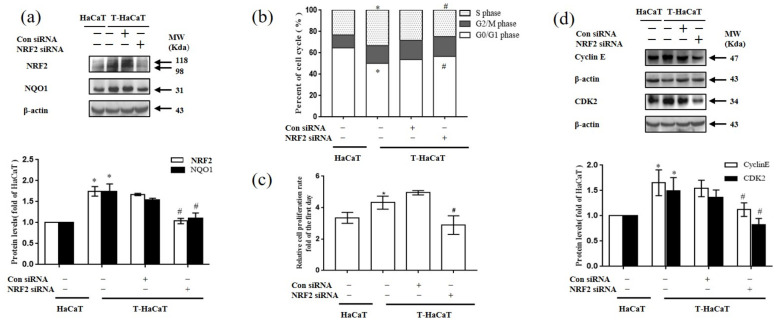
Effects of NRF2 on arsenite-induced malignant transformation of HaCaT cells. (**a**) Protein bands and relative protein levels of NRF2 and NQO1 in HaCaT cells and T-HaCaT cells with or without *NRF2* siRNA transfection. (**b**) Changes in the cell cycle. (**c**) Change in the cell proliferation rate. (**d**) Protein bands and relative protein levels of the cell-cycle-related proteins Cyclin E and CDK2. Error bars are the mean ± SD (*n* = 3). * Compared with HaCaT cells, *p* < 0.05; ^#^ Compared with control siRNA cells, *p* < 0.05.

Considering that the levels of NRF2 and NQO1 proteins in T-HaCaT cells were higher than those in the control, to understand more about the role in the arsenite-induced malignant transformation of HaCaT cells, *NRF2* was silenced by *NRF2* siRNA in arsenite-transformed T-HaCaT cells. After T-HaCaT cells were transfected with *NRF2* siRNA, the protein levels of NRF2 and NQO1 were significantly decreased in T-HaCaT cells compared with the con-siRNA transfected group (T-HaCaT (−)) (*p* < 0.05, Figure 3a). In addition, compared to the T-HaCaT (−) cells, the G0/G1 phase of the cells increased, whereas the S phase decreased after the T-HaCaT cells were transformed by *NRF2* siRNA (Figure 3b). It is suggested that silencing *NRF2* resulted in cell cycle stoppage. Levels of cyclic E-CDK2 proteins were reduced in transfected T-HaCaT cells compared to T-HaCaT (−) cells (*p* < 0.05, Figure 3d). Meanwhile, *NRF2* silencing blocked the proliferation of T-HaCaT cells (*p* < 0.05, Figure 3c).

**Figure 4:**
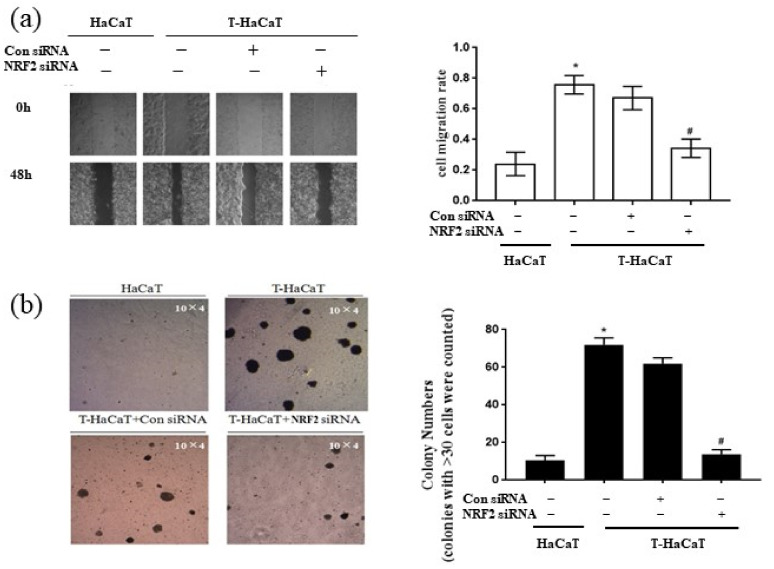
The cell migration rate and the colony number in T-HaCaT cells after transfection *NRF2*. (**a**) Wound-healing assay (Scheme 10 × 4) and the rate of migration. (**b**) Colony formation assay and colony numbers. Error bars are the mean ± SD (*n* = 3). * Compared with HaCaT cells, *p* < 0.05; ^#^ Compared with control siRNA cells, *p* < 0.05.

More importantly, silencing *NRF2* activity inhibited HaCaT cell migration, as detected by the wound-healing assay (*p* < 0.05, Figure 4a), and the anchorage-independent growth capacity was suppressed, as detected by colony formation in soft agar (*p* < 0.05, Figure 4b), which suggests that the malignant phenotype in HaCaT cells was relieved. These results demonstrated that NRF2 deficiency blocked the proliferation of T-HaCaT cells by blocking the change in the cell cycle, which suggested that NRF2 is a key factor in mediating cellular proliferation in arsenite-exposed HaCaT cells.

### 3.4. NQO1-Mediated Cellular Proliferation in Arsenite-Exposed HaCaT Cells

**Figure 5:**
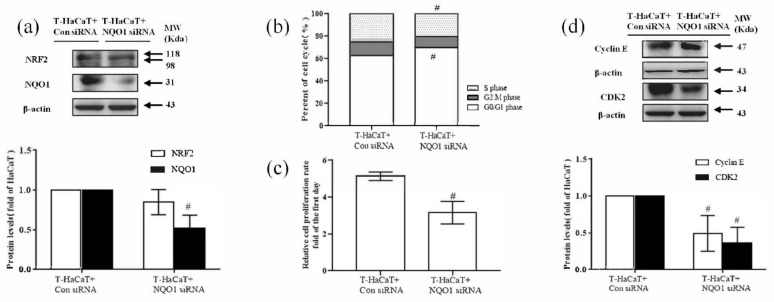
Effect of NQO1 on arsenite-induced malignant transformation of HaCaT cells. (**a**) Protein bands and relative protein levels of NRF2 and NQO1 in HaCaT cells and T-HaCaT cells with or without NQO1. (**b**) Changes in the cell cycle. (**c**) Change in the cell proliferation rate. (**d**) Protein bands and relative protein levels of cell cycle related protein Cyclin E and CDK2. Error bars are the mean ± SD (*n* = 3). ^#^ Compared with control siRNA cells, *p* < 0.05.

**Figure 6:**
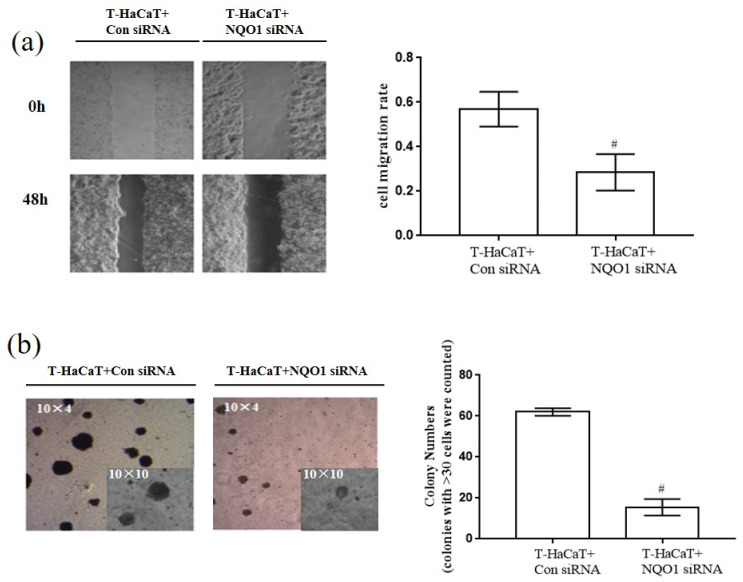
The cell migration rate and the colony number in T-HaCaT cells after transfection with *NQO1* siRNA. (**a**) Wound-healing assay (magnification factor: 10 × 4) and the rate of migration. (**b**) Colony formation assay and colony numbers. Error bars are the mean ± SD (*n* = 3). ^#^ Compared with control siRNA cells, *p* < 0.05.

To learn more about the role of NQO1 in the arsenide-induced malignant transformation of HaCaT cells, *NQO1* was silenced by *NQO1* siRNA in arsenite-transformed T-HaCaT cells. After *NQO1* siRNA transfection of T-HaCaT cells, the NQO1 protein level was significantly decreased in T-HaCaT cells, while the NRF2 protein level was not changed (*p* < 0.05, Figure 5a). The proportion of G0/G1 phase cells was significantly increased and S phase cells were decreased significantly after silencing *NQO1* (*p* < 0.05, Figure 5b), and the expression of the cell cycle-related proteins cyclin E and CDK2 was reduced after transfection (*p* < 0.05, Figure 5d). Furthermore, the proliferation of T-HaCaT cells was blocked (*p* < 0.05, Figure 5c), HaCaT cell migration was suppressed (*p* < 0.05, Figure 6a), and the anchorage-independent growth capacity of T-HaCaT cells was decreased (*p* < 0.05, Figure 6b) after blocking NQO1 expression with siRNA, which suggested that the malignant phenotype in HaCaT cells was relieved. These results demonstrated that NQO1 deficiency blocked the proliferation of T-HaCaT cells by blocking the change in the cell cycle, which suggested that, in addition to NRF2, NQO1 is also a key factor in cell proliferation in HaCaT cells exposed to arsenite.

## 4. Discussion

Long-term exposure to inorganic arsenic can lead to the occurrence of multisystem and multiorgan cancers [51,52]. Many studies have demonstrated the malignant transformation of normal cells after arsenite exposure and revealed the mechanism of arsenic-induced cancer [53,54,55]. For animal models of arsenic-induced skin cancer, which have not been constructed [56], HaCaT cells are widely used as an in vitro model to study the mechanism of arsenic-mediated skin cancer, especially in SCC [57,58,59]. Numerous studies have shown that exposure to a variety of exogenous chemicals may lead to the malignant transformation of HaCaT cells [60,61,62]. We found in an earlier study that HaCaT cells in soft agar exhibited high proliferation rates, increased MMP-9 secretion, and multicolony characteristics after being treated with 1.0 M sodium arsenite for more than 35 passages [44]. In the present study, we again verified that 1.0 µM sodium arsenite exposure can lead to a malignant phenotype in HaCaT cells by affecting the cell cycle of HaCaT cells (from the first passage) and increasing their proliferation rate from passage to passage (from passage 7) because sustaining proliferative signaling, evading growth suppressors, and replicative immortality are the hallmarks of cancer [31].

Furthermore, we explored the potential molecular mechanisms for promoting cellular proliferation for the carcinogenesis of arsenite. Many previous reports have suggested that the activation of NRF2 in tumor cells could affect proliferation [63,64] and accelerate the development of the disease [65,66,67,68], whose target gene *NQO1* also plays an important role in cellular proliferation and is overexpressed in many cancer tissues [41,69,70]. Indeed, our past studies have found that high levels of NRF2 may induce arsenite-induced tumors [44,46]; in this study, we observed that either high levels of NRF2 (after seven passages) or NQO1 (after 21 passages) were caused by arsenite. Notably, the in vitro UV irradiation of melanocytes has been demonstrated to induce the activation of NRF2 and its target genes, including *NQO1* [71]. Consistent with our results, NQO1 promotes an aggressive phenotype in hepatocellular carcinoma by amplifying ERK-NRF2 signaling [72]; immunohistochemical analysis showed that the protein levels of NRF2 and NQO1 were higher in carcinoma tissues than in benign follicular adenomas and hyperplastic nodules [73]. Overall, the NRF2/NQO1 pathway is important in response to environmental toxins and may be a tumor suppressor [74]. In addition, the results of the study also indicated that arsenite can regulate the protein expression levels of cyclin E and CDK2, which are also associated with cell proliferation and malignant transformation. This finding explains the cell cycle progression from the G0/G1 to S phase observed in this study. Cyclin E is an essential component of the nuclear cell cycle machinery. In mammalian cells, two types of cyclin E activate CDK2 and initiate cell cycle progression by phosphorylating various cellular proteins [75]. Cyclin E appears at the end of the G1 phase, which can facilitate and shorten the transition from the G/S phase [76]. Cyclin E binds to CDK2 at the end of stage G1, activates CDK2 activity, and promotes the expression of genes related to DNA replication, thereby initiating cellular DNA replication [77]. Lee et al. found that the fargesin-induced colony growth inhibition of colon cancer cells was mediated by suppression of the cyclin-dependent kinase 2 (CDK2)/cyclin E signaling axis by the upregulation of p21WAF1/Cip1, resulting in G1-phase cell cycle accumulation in a dose-dependent manner [78]. Liang et al. demonstrated that the administration of cyclin E siRNA could inhibit breast tumor growth in nude mice [79]. Therefore, we proposed that arsenite can induce the malignant transformation of HaCaT cells, affect cell cycle progression, and promote cell proliferation. The phase shift of the cell cycle can be adjusted by the G0/G1 and S phase structural ratio by increasing the expression of Cyclin E-CDK2.

In addition, the upstream–downstream relationship between NRF2/NQO1 and cyclin E-CDK2 in promoting cell proliferation and subsequent malignant transformation caused by arsenite drew our attention. To clarify this problem, we silenced the 35 passages of T-HaCaT cells by transfection with *NRF2* siRNA and *NQO1* siRNA, a kind of double-stranded RNA with a length of 20 to 25 nucleotides, by interfering with the post transcriptional degradation of mRNA expressing specific genes with complementary nucleotide sequences, thus preventing translation. We found that in addition to downregulating NQO1 expression, the silencing of *NRF2* by siRNA interference reduced the cell proliferation rate and cell cycle-related proteins Cyclin E and CDK2 and ultimately decreased the migration rate and colony-forming capacity of T-HaCaT cells. This result corresponds to Homma et al. [66]. They found that the knockout of *NRF2* in A549 and NCI-H292 cells caused cell cycle arrest in the G1 phase. Furthermore, other in vitro experiments showed that a lack of *NRF2* prevented cell cycle progression and slowed cell proliferation [80,81]. Moreover, there are few reports of the relationship between NQO1 and cyclin E-CDK2. We further observed the same situation as *NRF2* and *NQO1* silencing resulted in decreased cyclin E and CDK2 protein levels, arrested cell cycle progression from the G0/G1 to S phase, and restricted cell proliferation and malignant phenotypes in arsenite-transformed cells, which were characterized by reduced colonization and limited migration. This result is consistent with our previous report on HBC cells [48]. From this, we conclude that a new common pathway of arsenic carcinogenesis has been identified.

NRF2 and NQO1 are generally recognized to play important roles in cancer prevention and treatment [82,83,84,85], so they can be targeted for skin cancer. Researchers searched for molecules able to intentionally activate NRF2 because it has been shown to be a way to prevent skin cancer [86,87,88]. As the activation of NRF2 in cancer is two-sided [36,89], its functions are more far-reaching than originally envisioned, which presents new challenges and opportunities for targeting NRF2 and NQO1 in skin cancer prevention and treatment [90].

Unfortunately, we do not yet know the mechanism by which the NRF2/NQO1 pathway regulates cyclin E-CDK2; in addition, we performed all the experiments on a single cell line, and we have not yet validated our results based on animal models by using cell-derived xenografts, which are our research plans for the future. It must be acknowledged that the mechanism of SCC is complex and must therefore be taken into account holistically in the interpretation of the mechanism and future treatment. Moreover, a genomic approach is necessary for determining DNA mutations caused by arsenite treatment in future studies, which is important to understand whether arsenite is a carcinogenicity promoter or initiator.

## 5. Conclusions

**Figure 7:**
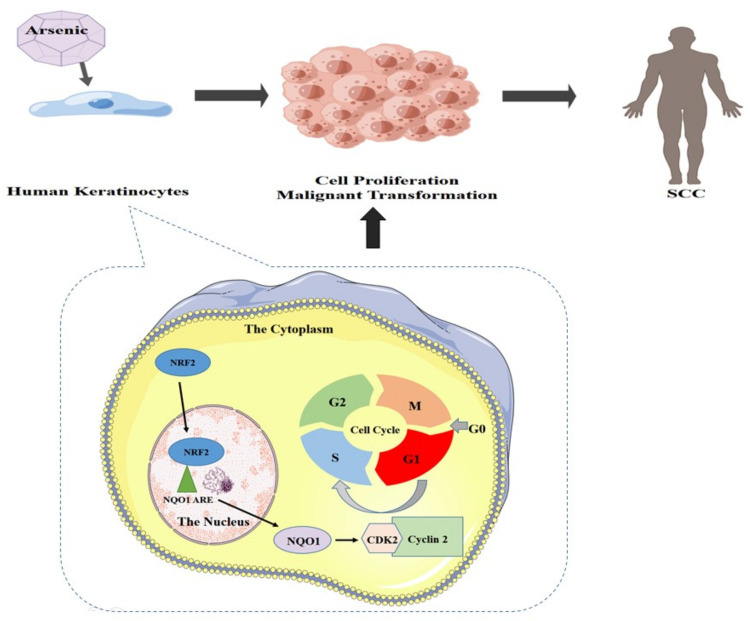
Proposed model by which NRF2 regulates NQO1 to promote proliferation in arsenite-induced malignant transformation.

In conclusion, we demonstrated that NRF2/NQO1 plays an important role in the arsenite-induced malignant transformation of cells. Increased regulation of NRF2/NQO1 increases cyclin E-cdk2 expression and promotes arsenite-induced disruption of the HaCaT cell cycle from the G0/G1 to S phase, which leads to cell proliferation and malignancy. In summary, NRF2 has been shown to regulate NQO1, and both contribute to the transformation of arsenite-induced cell malignancy (Figure 7). Our research provides important information about the molecular mechanisms of arsenic-induced SCC. We found that using siRNA to silence *NRF2* and *NQO1* led to a significant decrease in protein expression in genes downstream of NRF2 (NQO1) and Cyclin E-CDK2. Therefore, these results suggest that the silencing of *NRF2* and *NQO1* inhibits the growth of HaCaT cells and that arsenite-induced malignant phenotypes are alleviated. In summary, the findings from this study can assist in further understanding the health effect mechanism of arsenic pollution and facilitate future developments of new arsenic and other similar environmental factors (e.g., UV radiation)-induced SCC treatment and prevention.

## Data Availability

Not applicable.

## Abbreviations

ANOVA	analysis of variance
ARE	antioxidant response element
CDK2	Cyclin-dependent kinase 2
DMEM	Dulbecco’s modified Eagle’s medium
FBS	Fetal bovine serum
HaCaT	immortalized human keratinocyte cells
Keap1	Kelch-like ECH-associated protein 1
NaAsO_2_	sodium arsenite
NRF2	nuclear factor-erythroid-2 p45-related factor 2
NQO-1	NAD(P) H: Quinine oxidoreductase 1
NADPH	nicotinamide adenine dinucleotide phosphate
PBS	phosphate-buffered saline
PI	Propidium iodide
ROS	reactive oxygen species
T-HaCaT	arsenite-transformed HaCaT cells
HBE	human bronchial epithelial
MC	skin melanoma
NMSC	non-melanoma skin cancer
BCC	basal cell carcinoma
SCC	squamous cell carcinoma
UV	ultraviolet radiation
siRNA	small interfering RNA
